# Lennox-Gastaut Syndrome: In a Nutshell

**DOI:** 10.7759/cureus.3134

**Published:** 2018-08-13

**Authors:** Muhammad Umair Jahngir, Malik Qistas Ahmad, Memoona Jahangir

**Affiliations:** 1 Neurology, University of Missouri Healthcare, Columbia, USA; 2 Hematology-Oncology, University of Arizona Cancer Center, Tucson, USA; 3 Internal Medicine, Punjab Medical College Allied Hospital Faisalabad, Faisalabad, PAK

**Keywords:** childhood, epilepsy, encephalopathy, intellectual disability

## Abstract

Lennox-Gastaut syndrome is one of the rare childhood-onset epileptic encephalopathies, characterized by multiple type seizure disorder, the typical pattern on electroencephalogram and intellectual disability. Tonic-type seizures are most commonly seen in these patients. Behavioral disturbances and cognitive decline are gradual-onset and last long after the first episode of epileptiform activity. In most cases, there is some identifiable cause that has led to the clinical presentation of the patient. Various pharmacological and surgical procedures have been proposed for the treatment of Lennox-Gastaut syndrome and many more to come in the very near future to overcome the drug resistance and to avoid the patient forming a life-long dependency.

## Introduction and background

Lennox-Gastaut syndrome (LGS) is one of the eight syndromes under epileptic encephalopathy, as reported by the International League Against Epilepsy (ILAE) task force [1-2]. The term “epileptic encephalopathy” is defined as a progressive decline of cerebral function and cognition along with behavioral regression or deterioration, caused by epileptogenic (ictal and electrical) activity during the period of brain maturation [1-4]. Lennox-Gastaut syndrome was first named by Lennox as “Petit mal variant,” which was later named after him in 1966 by Gastaut and his coworkers as Lennox syndrome. This was referred to as a childhood-onset tonic and the absence type of seizure [3]. Lennox-Gastaut syndrome accounts for approximately 1%–10% of childhood epilepsies. The clinical picture of LGS is the triad of antiepileptic-resistant seizure disorder, characteristic abnormality on electroencephalogram (EEG), and cognitive dysfunction. The epileptiform abnormalities contribute to gradual-onset intellectual disabilities along with psychiatric comorbidities like anxiety, depression, and behavioral abnormalities. All the three criteria must not be present at the time of the onset of seizure and diagnosis is established after following up with the patient for several years [5].

Lennox-Gastaut syndrome is a typical childhood-onset, severe epileptic encephalopathy associated with a serious intellectual disability in 20%-60% patients at the time of the onset of seizures, the proportion of which will increase to 75%-95% at five years after the onset of seizures. Though some cases with normal intellect and behavior have also been reported. The specific electroencephalographic pattern varies from either bursts of slow spike-wave complexes or generalized paroxysmal fast activity. Slow spike-wave complexes (<2.5 Hz) are not considered to be pathognomonic for Lenox-Gastaut syndrome, but some experts have found generalized paroxysmal fast activity on electroencephalogram to be an essential criterion for diagnosing Lennox-Gastaut syndrome [5].

## Review

Epidemiology

The prevalence of Lennox-Gastaut syndrome is 1%-2% of the epileptic patients and 1%-10% (mean 4%) of the childhood epilepsies [3]. The age of onset ranged from the tenth day of life to nine years of age, with the mean age of onset of 35.3 months [5]. The peak age of the onset in patients with the cryptogenic Lennox-Gastaut syndrome is lower than in those with a disease due to some identifiable etiology [1]. Lennox-Gastaut was once defined as any multiple seizure type epilepsy in children less than 11 years old. However late-onset syndrome has also been found in the literature [3]. At the time of onset, all patients suffer multiple types of seizures, most commonly tonic seizures, followed by generalized tonic-clonic seizures. Almost all the patients are on two to six antiepileptic drugs (mean 3.4). Of the patients, 28% have been found to be seizure-free after using these drugs for at least 1.5 years [5].

According to prospective studies, 7%-17% of patients with a severe intellectual disability have Lennox-Gastaut syndrome. Lennox-Gastaut syndrome is found to be more prevalent in males than in females, for some undefined reason, with the male to female ratio being 1:6 (relative risk, 5.31) [1,3]. The proportion of patients with Lennox-Gastaut syndrome in European countries like Spain, Estonia, Italy, and Finland is found to be consistent across the studied populations and has demonstrated a similarity to that in the United States [1].

Etiology

In general, the causes of Lennox-Gastaut syndrome is divided into two broader groups [3,5]:

1. Identifiable causes

2. Cryptogenic (non-identifiable) causes

Identifiable causes contribute to 67% to 75% of the patients with Lennox-Gastaut syndrome. These include brain damage (e.g., head trauma), perinatal complications (e.g., birth asphyxia, intrauterine growth retardation, kernicterus), congenital central nervous system malformations (e.g., tuberous sclerosis), infections (e.g., meningitis, sepsis), or metabolic disorders. A retrospective study has shown that birth complications contribute to 25% of Lennox-Gastaut syndrome while patients with this disease have histories of central nervous system infection and head trauma in 3.7% and 1% of the cases, respectively [3].

Cryptogenic Lennox-Gastaut syndrome was found in the remaining 25% to 35% of patients. Many studies have tried to explain the mystery behind it by coupling the phenotypic description of the epileptic patients and their parents, with genetic studies of the pedigree. It has been seen in 16%-20% of the patients with diagnosed West syndrome/infantile spasms that finally turned out to be Lennox-Gastaut syndrome [3].

Copy number variants have been found in 2.9%-19% of the patients with Lennox-Gastaut syndrome. The mutation of other genes involved in human brain development (e.g., the forkhead box G1 (FOXG1), chromodomain-helicase-DNA-binding protein 2 (CHD2) genes) and the gene for presynaptic protein dynamin 1 (DNM 1) have also been found to be associated with this syndrome. The genetic heterogeneity of this syndrome identifies it to be one of the manifestations of other genetic disorders, rather being an independent entity [3,5].

Clinical presentation

The clinical picture is a triad of the following:

1. Seizure disorder

2. A characteristic pattern on electroencephalogram

3. Cognitive impairment

The tonic type of seizure is seen in all patients with Lennox-Gastaut syndrome but may not be present at the time of its onset. Atypical absence seizures (with gradual onset and termination) are the second-most common type of epileptic activity seen in these patients, but it is difficult to diagnose clinically in patients with diminished cognition. Prolonged atypical absences are seen in 66% of the patients with altered consciousness, which is periodically interrupted by episodes of tonic seizures. These non-convulsive episodes may last for hours to weeks in severe cases, clinically named as ‘non-convulsive status epilepticus’ [3].

Atonic and myoclonic seizures have also been recorded in patients with Lennox-Gastaut syndrome. Drop attacks are also common (more than 50%) in these patients, but is not a pathognomonic clinical manifestation. Other types of seizures more commonly seen in later stages of the disease include focal seizures, generalized tonic-clonic seizures, or unilateral clonic seizures [3].

Lennox-Gastaut syndrome seems to be the result of a central nervous system network dysfunction and is sometimes referred to as “secondary network epilepsy.” The mostly seen EEG patterns are slow spike-wave complexes (SSW) and generalized paroxysmal fast activity (GPFA). These EEG patterns were studied while coupling them with functional magnetic resonance imaging (fMRI) findings, which revealed the fact that patients with generalized paroxysmal fast activity on EEG had increased blood oxygen level-dependent (BOLD) signals in cortical “association” areas, the brain stem, basal ganglia, and thalamus, while patients with slow spike-wave pattern on EEG had primarily decreased BOLD signals in “primary cortical areas.” This concludes that GPFA is the result of more diffuse activation of cortical and subcortical neuronal networks. However, cortical and subcortical activations and deactivations are associated with SSW complexes [2-3]. Single-photon emission computed tomography (SPECT) during the peri-ictal phase in patients with Lennox-Gastaut syndrome with tonic seizures has shown the activity in the bilateral frontal and parietal association areas and the pons [2].

According to a case-control study, what increases the odds of having intellectual disability in the patients with Lennox-Gastaut syndrome include a history of non-convulsive status epilepticus, a diagnosed case of West syndrome, any identifiable etiology of having epilepsy, and an early age of onset. Cognitive decline is secondary to epilepsy itself, like all other epileptic encephalopathies or may be due to abnormal neuronal connections and the side effects of medications [3].

Diagnosis

The following test can be considered to diagnose Lennox-Gastaut syndrome [1,3-4];

1. Complete blood panel (rule out the metabolic causes)

2. Magnetic resonance imaging brain

3. Electroencephalogram

4. Genotyping (chromosomal array, Sanger sequencing, next-generation sequencing panels)

Differential diagnosis

Other medical diagnoses to be kept in mind include the following infantile/childhood epileptic encephalopathies (Table 1) [1,3,6-7]:

**Table 1:** Differential diagnosis of Lennox-Gastaut syndrome

Syndrome	Age of onset	Seizure type	EEG findings
Ohtahara Syndrome (epileptic encephalopathy)	First three months (usually within first 10 days of birth)	Tonic/clonic, clonic, myoclonic, atonic, absences, partial, complex partial (with or without secondary generalization)	Burst and suppression pattern during both waking and sleeping states.
West Syndrome (epileptic encephalopathy)	4–6 months	Epileptic spasms	Hypsarrthymia
Dravet Syndrome (severe myoclonic epilepsy)	First year	Focal or secondarily generalized with fever in infancy; myoclonus after 1 year of age	Often normal at onset; generalized spikes/polyspikes activated with a photic stimulation
Pseudo-Lennox-Gastaut Syndrome (atypical benign partial epilepsy)	Early childhood	Atypical absence, myoclonus, atonic, and focal seizures	Rolandic sharp waves, multifocal sharp waves, electrical status epilepticus in sleep
Doose Syndrome (myoclonic-atonic epilepsy)	Early childhood	Myoclonic-atonic, myoclonus, and atypical absence	2–3 Hz generalized spike-waves, photo-paroxysmal response
Electrical Status Epilepticus during Slow Sleep (ESES)	Two months - 12 years (peak age 4 and 5 years)	Unilateral or bilateral clonic, generalized tonic-clonic, absences, complex partial seizures, with or without drop attacks	Spikes and waves occurring almost continuously during slow sleep (subclinical)
Acquired Epileptic Aphasia Landau-Kleffner Syndrome (LKS)	18 months – 13 years	Generalized tonic-clonic seizures	Paroxysmal electroencephalographic changes and little or no language development
Juvenile Myoclonic Epilepsy	Adolescence	Myoclonic jerks with or without generalized tonic-clonic seizures and/or absence seizures	3-5 Hz generalized spike waves and polyspikes with normal background activity

Other differential diagnoses to be considered are [8]:

- Toxic encephalopathy - Metabolic diseases (e.g., Glut-1 deficiency syndrome, pyruvate dehydrogenase deficiency)

Treatment

Various pharmacological and surgical options are now available to treat these patients. Most patients with epileptic encephalopathies are resistant to anti-epileptic drugs and most of them are on more than two medications for seizure control [3]. *Anti-epileptics*

Valproate, lamotrigine, and topiramate are declared to be the first line. According to randomized control trials, other anti-epileptics reported to be effective are clobazam, felbamate, and rufinamide (Table 2) [2-3].

**Table 2:** Anti-epileptic drugs and their application * Recently approved anti-epileptic drugs for Lennox-Gastaut syndrome

Drugs	Important side effects	Remarks	Starting doses	Maintenance dose
Valproate	Hepatotoxicity, pancreatitis, drug interactions	Most effective for myoclonic, atypical absence, and atonic seizures	Started at 7–10 mg/kg/day PO, weekly increased by 5 mg/kg/day as tolerated and necessary	60 mg/kg/day or 3000 mg/day
Lamotrigine	Skin reactions, drowsiness, nausea, anorexia, headache, and ataxia, exacerbate myoclonic seizures	Effective for tonic-clonic seizures and drop attacks	Age <12 years: 0.3 mg/kg/day in 1 or 2 divided doses for the first two weeks. Age >12 years: 25 mg/day for the first two weeks. Increases by up to 50 mg/day every 1-2 weeks	300 mg/day in two divided doses
Topiramate*	Anorexia, weight loss, renal stones, and slowing of cognition		Age 2-10 years: 0.5–1 mg/kg/day for 1–2 weeks, increases gradually by 0.5–1 mg/kg/day every 1–2 weeks. Age >10 years: 25 mg nightly for one week, increases weekly by 25 mg/day over 2-4 weeks	Age < 16 years: 18 mg/kg/day. Age >16 years: 600 mg/day, increases up to 1600 mg/day
Felbamate*	Aplastic anemia, liver failure	Safety and efficacy not established for age <14 years	1200 mg/day divided every 6-8 hr, increases two weekly by 600 mg, up to 2400 mg/day	Maximum dose is 3600 mg/day
Clobazam*	Sedation	Adjunctive treatment, decreases the frequency of drop attacks Approved for two or more year-old patient	Weight <30 kg: 0.25 mg/kg/day in two divided doses. Increased by 5–15 mg every 5 days until seizures are controlled. Weight >30 kg: 5-10 mg/day in 1-2 doses	Weight <30 kg: 1 mg/kg/day. Weight >30 kg: 80 mg/kg/day
Rufinamide*	Somnolence, vomiting, and weight loss Contraindicated in patients with familial short QT syndrome	Adjunctive treatment of seizures in 4 or more year-old patient Effective for drop attacks	Age 4 - 15 years: 10 mg/kg/day, in two equally divided doses, and increases by 10 mg/kg every other day up to target doses. Age >15 years: 400 - 800 mg/day, in two equally divided doses and increased by 400 - 800 mg/kg every other day	Age 4 - 15 years: 45 mg/kg/day or 3200 mg/day, whichever is less. Age > 15 years: 3200 mg/day

It is a general principle to prescribe the least possible number of drugs at a time, at the lowest possible doses. If the first drug fails, convert him to another drug, but if the second drug is also not effective, add a second agent to the existing regimen [3].

Status epilepticus is a medical emergency and should be treated with benzodiazepines like other patients with prolonged seizures. Non-convulsive status epilepticus are also responsive to corticosteroids and/or ketogenic diet [8].

Ketogenic Diet

It is the leftover option for those patients who are poorly responding to medical treatment [1]. It is found to be effective in childhood refractory seizures in specific genetic disorders, e.g., Glut-1 deficiency syndrome [2]. The common side effects of the ketogenic diet are constipation, vomiting, abdominal pain, apathy, increase appetite, hypercholesterolemia, mineral deficiencies, acidosis, and growth retardation. Some studies have shown that the use of a low glycemic index diet and modified Atkins diet (containing nuts/seeds, fruits, or dairy products) are also effective in these patients [3].

Surgery

Surgical options for Lennox-Gastaut syndrome include corpus callosotomy, vagus nerve stimulation, and focal cortical resection [1].

Corpus callosotomy: The corpus callosum consists of the ‘rostrum,’ ‘genu,’ ‘body,’ and ‘splenium’ (anterior to posterior). According to its topographical representation, the resection of the anterior 4/5th is sufficient to produce effective results in these patients, though a 10% lower response rate as compared to those with total resection of the corpus callosum. Keeping the splenium preserved will preserve some of the fibers for interhemispheric perceptual information to transfer and to lessen the complications of ‘disconnection syndrome’ [3].

Vagus nerve stimulation: The exact mechanism of vagus nerve stimulation in patients with epilepsy is yet to be known. It has been suggested it might interrupt the synchronicity of electrical activity or might cause changes in metabolism or blood flow to various cortical and sub-cortical areas of the brain. It is reserved for patients with medically refractory seizures where surgery is not the option. It is effective in all types of seizures and adverse effects are much less as compared to corpus callosotomy. The most common side effects are hoarseness of voice, dysphagia, dyspnea, and coughing [3].

Cortectomy/lobar dissection: A selective dissection of the cortex may produce immediate and spectacular results in disabling seizures [8]. A meta-analysis has shown significantly better results after callosotomy as compared to those after vagus nerve stimulation [9].

Other options are gamma knife callosotomy, deep brain stimulation, and multiple subpial transactions [8].

Prognosis

Various prospective studies have shown that features of the typical Lennox-Gastaut syndrome will evolve over time, and it will be difficult to identify if it remains undiagnosed in childhood. The variety and frequency of seizures decrease over time, and so does their severity. Generalized paroxysmal fast activity on EEG will typically persist in adulthood, while slow spike-wave complexes will remain in a minority of the patients. Cognitive and behavioral disturbances will remain in most of the patients with Lennox-Gastaut syndrome as an adult [3]. The long-term outcome of the disease is variable, from normally functioning individuals to severe mental retardation and treatment-resistant seizures in 47%-76% of the patients who need special home or institutional care [1].

Advances

Drug resistance and an increased understanding of the disease process has forced us to discover the novel methods of dealing with the disease. Lacosamide has been found to be effective for focal and tonic-clonic seizures and is used as adjunctive treatment. What is most concerning about lacosamide use is that it may exacerbate tonic seizures in Lennox-Gastaut syndrome patients [4].

Other anti-epileptic drugs, which are suspected to be beneficial in these patients, include carisbamate (increases seizure threshold), fluorofelbamate (dicarbamate), ganaxolone (GABA-A receptor modulator), seletracetam (levetiracetam derivatives), and remacemide (NMDA receptor blocker). More clinical trials are needed to define the drug safety and their maximal tolerable doses [8]. Steroids, along with antiepileptic drugs in these patients, have shown promising results [3,8]. A global review has identified intravenous immunoglobulins (IVIG) effective in various infantile and childhood epilepsies, including those with Lennox-Gastaut syndrome [3].

Neuroprotection is an emerging concept in the treatment of Lennox-Gastaut syndrome patients. Some trials have shown the efficacy of melatonin and NMDA receptor antagonists in neuronal protection and the control of epilepsy. Gene therapy is also a fascinating option that needs to be pondered on in cases of drug-resistant epilepsies and in those patients who are not suitable candidates for surgery [8].

The overall outcome of two, double-blinded, well-controlled trials has shown that cannabinoids are effective in patients with Lennox-Gastaut syndrome as compared to the placebo at the dose of 10-20 mg/kg/day [10].

## Conclusions

Lennox-Gastaut syndrome is a clinical triad of drug-resistant seizures, pathognomonic electroencephalographic patterns, along with mildly to severely decreased intelligence quotient (IQ). The etiology of this syndrome is broadly divided into identifiable and non-identifiable causes. Identifiable causes comprise the major portion of this classification and include various diseases that are structural, genetic, or metabolic in origin. Patients with Lennox-Gastaut syndrome, like all other epileptiform encephalopathies, are multi-drug resistant. Many experts think valproate, lamotrigine, and topiramate are the first-line drugs while managing this syndrome. Steroids, immunomodulation, or dietary restrictions have proved their roles in the treatment of these patients. Severely damaging seizures that are not responsive to medical treatment are treated by a complete or partial resection of corpus callosum fibers. In those who are not ideal candidates for surgery, vagus nerve stimulation is providing promising results. Overall, the prognosis of Lennox-Gastaut syndrome is debilitating, both for the patient and the family of the patient.
